# Complete mitochondrial genome of *Manispentadactylapentadactyla* (Mammalia: Pholidota), an endemic subspecies of Chinese pangolin: mitogenome characterisation and phylogenetic implications

**DOI:** 10.3897/BDJ.9.e77961

**Published:** 2021-12-29

**Authors:** Nick Ching-Min Sun, Chi-Chun Huang, Yu-Wei Tseng, Tulshi Laxmi Suwal, Meng-Jou Chi, Nian-Hong Jang-Liaw, Kuo-Hsiang Hung

**Affiliations:** 1 Department of Entomology, National Chung Hsing University, Taichung, Taiwan Department of Entomology, National Chung Hsing University Taichung Taiwan; 2 IUCN SSC Pangolin Specialist Group, Zoological Society of London, London, United Kingdom IUCN SSC Pangolin Specialist Group, Zoological Society of London London United Kingdom; 3 Taiwan Endemic Species Research Institute, Nantou, Taiwan Taiwan Endemic Species Research Institute Nantou Taiwan; 4 Graduate Institute of Bioresources, National Pingtung University of Science and Technology, Pingtung, Taiwan Graduate Institute of Bioresources, National Pingtung University of Science and Technology Pingtung Taiwan; 5 Small Mammals Conservation and Research Foundation, Kathmandu, Nepal Small Mammals Conservation and Research Foundation Kathmandu Nepal; 6 Department of Tropical Agriculture and International Cooperation, National Pingtung University of Science and Technology, Pingtung, Taiwan Department of Tropical Agriculture and International Cooperation, National Pingtung University of Science and Technology Pingtung Taiwan; 7 WildOne Wildlife Conservation Association, Taitung, Taiwan WildOne Wildlife Conservation Association Taitung Taiwan; 8 Taipei Zoo, Taipei, Taiwan Taipei Zoo Taipei Taiwan; 9 Biodiversity Research Center, National Pingtung University of Science and Technology, Pingtung, Taiwan Biodiversity Research Center, National Pingtung University of Science and Technology Pingtung Taiwan

**Keywords:** critically endangered, *Manispentadactyla*, mitogenome, phylogeny

## Abstract

The Chinese pangolin *Manispentadactyla* is critically endangered because of over-exploitation and illegal trafficking and includes three subspecies. However, the taxonomic status of the three subspecies of the Chinese pangolin has not been well resolved, which impedes regional conservation and illegal trade traces. In this study, the complete mitogenome sequence of *M.p.pentadactyla*, an endemic subspecies of the Chinese pangolin in Taiwan, was determined. The complete mitogenome of *M.p.pentadactyla* is 16,570 base pairs (bp) in length with 13 protein-coding genes (PCG), 23 transfer RNAs (tRNAs), two ribosomal RNAs and a 1164 bp control region. The overall base composition of the genome showed a slight A + T bias (59.9%), positive AT skew (0.1515) and negative GC skew (-0.3406), which is similar to that of other pangolins. All PCGs started with a typical ATN codon and all tRNAs were typical cloverleaf-shaped secondary structures, except for tRNA-Ser^(GCU)^. Phylogenetic analysis indicated a monophyletic relationship for *M.p.pentadactyla* and *M.p.aurita* and was monophyletic for *M.p.pentadactyla*, but paraphyletic for *M.p.aurita*. The paraphyly of *M.p.aurita* resulted from an incomplete lineage sorting. This study enriched the mitogenome database of the Chinese pangolin and the molecular information obtained should be very useful for future research on mitogenome evolution and genetic diversification in *M.pentadactyla*.

## Introduction

Pangolins (Mammalia: Pholidota) are scaly-bodied mammals that inhabit a wide range of ecosystems, including secondary subtropical rainforests, bamboo forests, broadleaf forests, savanna woodlands, grasslands and agricultural landscapes in Africa and Asia (Chao et al. 2020). Pangolins are almost exclusively termite- and ant‐eating species, consuming all life stages of their prey, including eggs, larvae, pupae and adults. An individual pangolin consumes several million prey items each year (Harrison 1961, Pietersen et al. 2016, Lee et al. 2017, Sun et al. 2020). Thus, pangolins provide an essential ecological function in maintaining the balance of ecosystems and biodiversity by regulating the number of ants and termites (Del Toro et al. 2012, Chao et al. 2020).

The Chinese pangolin *Manispentadactyla* Linnaeus, 1758 (Mammalia: Pholidota), is widely found in East Asia, northern Southeast Asia and parts of South Asia, including Nepal, North India, Bhutan, Bangladesh, Myanmar and the northern Indochinese Peninsula, throughout most regions to the south of the Yangtze River in China and the Islands of Hainan and Taiwan (Wu et al. 2020). Over the past several decades, the Chinese pangolins have been over-exploited because of the massive demand for traditional medicines, health supplements, leather and ornaments (Challender et al. 2014). The Chinese pangolin is categorised as ‘Critically Endangered’ on the IUCN Red List of Threatened Species (Challender et al. 2019) and is also listed in Appendix I of the Convention on International Trade in Endangered Species of Wild Fauna and Flora (CITES 2021).

Three subspecies of the Chinese pangolin have been identified in some reports based on morphological traits (Allen 1906, Allen 1938). The type specimen of *M.pentadactyla* was collected from Taiwan and the nominal subspecies, *M.p.pentadactyla* Linnaeus, 1758, refers to the Taiwanese population (Sun et al. 2019). The other two subspecies are *M.p.aurita* Hodgson, 1836, found in mainland Asia and *M.p.pusilla* Allen, 1906 on the Hainan Island in China (Wu et al. 2020). As pangolins may display a greater intraspecific molecular variation than other mammals, assessment within a total evidence framework is required (Pietersen and Challender 2020). Thus, the subspecies status of the Chinese pangolin remains to be clarified with genetic evidence, including the nominal Formosan pangolin in Taiwan. However, the taxonomic status of all three subspecies has not been well resolved by genetic evidence (Wu et al. 2007, Hua et al. 2020, Wu et al. 2020). This impedes regional conservation and potential reintroduction, as well as illegal trade traces and law enforcement.

An increase in pangolin numbers has been documented in many areas throughout Taiwan (Wu et al. 2007, Sun et al. 2019). In addition, the genetic structure of *M.p.pentadactyla* in a local population in eastern Taiwan has been assessed (Sun et al. 2020). In this study, we further determined the complete mitogenome of *M.p.pentadactyla* and constructed a phylogenetic tree of all eight pangolin species in Manidae. Furthermore, comparative mitochondrial genome analysis was carried out to identify similarities and differences between the subspecies of the Chinese pangolin.

## Material and methods

### Sample collection and genomic DNA extraction

The sample of *M.p.pentadactyla* was obtained from eastern Taiwan (23°7'N, 121°12'E), with permission from the Taiwan Forestry Bureau (permit number 1101607633 as required by the Wildlife Conservation Act 2013). The tissue was preserved in 70% ethanol for subsequent DNA extraction. Genomic DNA was extracted using a phenol-chloroform protocol (Sambrook and Russell 2001). The spectrophotometer NanoDrop 2000 (Thermo Fisher Scientific, MA, USA) was used to measure genomic DNA concentration.

### Genome sequence assembly and analyses

Before genome sequencing, the quality/quantity of DNA samples were assessed using the Agilent Genomic DNA ScreenTape assay in conjunction with the 4200 TapeStation system (Agilent Technologies). The 10 ug of total DNA was sonicated using a Covaris M220 Focused-ultrasonicator to a size ranging from 400 to 500 bp. Subsequently, genomic DNA was used for library preparation using the Illumina Truseq DNA Sample Preparation Kit (Illumina, San Diego, USA), following the manufacturers’ preparation protocol. Genome sequencing was performed using the Illumina HiSeq platform for PE 2 × 150 bp sequencing. The raw sequences were filtered to obtain qualified reads using FASTP v.0.20 (Chen et al. 2018) and FLASH v.1.2 was used to merge paired-end reads (Magoč and Salzberg 2011). The complete circular mitochondrial genome of *M.p.pentadactyla* was assembled de novo with MitoFinder v.1.3 (Allio et al. 2020). Approximately 32.34 GB of clean data were obtained, yielding about 2192-fold depth of coverage of the mitogenome.

### Mitogenome analyses

The assembled mitochondrial genome was annotated using the MITOS2 web server to predict the location of protein-coding genes (PCGs), transfer RNAs (tRNAs), ribosomal RNAs (rRNAs) and putative secondary structures of tRNAs (http://mitos2.bioinf.uni-leipzig.de/index.py) (Donath et al. 2019). The sequence with annotated features has been deposited in GenBank (Accession Number MZ868226) and a circular map of the mitochondria was generated using OGDRAW v.1.3.1 (Lohse et al. 2007). All PCGs codon usage and nucleotide frequencies were obtained using the Molecular Evolutionary Genetics Analysis software MEGA version X (Kumar et al. 2018). We also calculated AT skew and GC skew for full mitochondria and genes using the formula: AT skew = (A–T) / (A+T) and GC skew = (G–C) / (G+C) (Perna and Koeher 1995).

### Phylogenetic analyses

Phylogenetic relationships were constructed based on the full mitogenome and 13 PCG sequences of *M.p.pentadactyla*, *M.p.aurita* and other seven closely related pangolins (Suppl. material 1). Sequence alignment was conducted using the MAFFT online server (Katoh et al. 2019). The phylogeny with Maximum Likelihood (ML) method was performed in IQ-TREE v.2.1.3 (Nguyen et al. 2015). The GTR + F + I + G4 model and an ultrafast bootstrap approximation algorithm were selected (Minh et al. 2013).

## Results and Discussion

### Characteristics and composition of mitogenome

**Figure 1.**
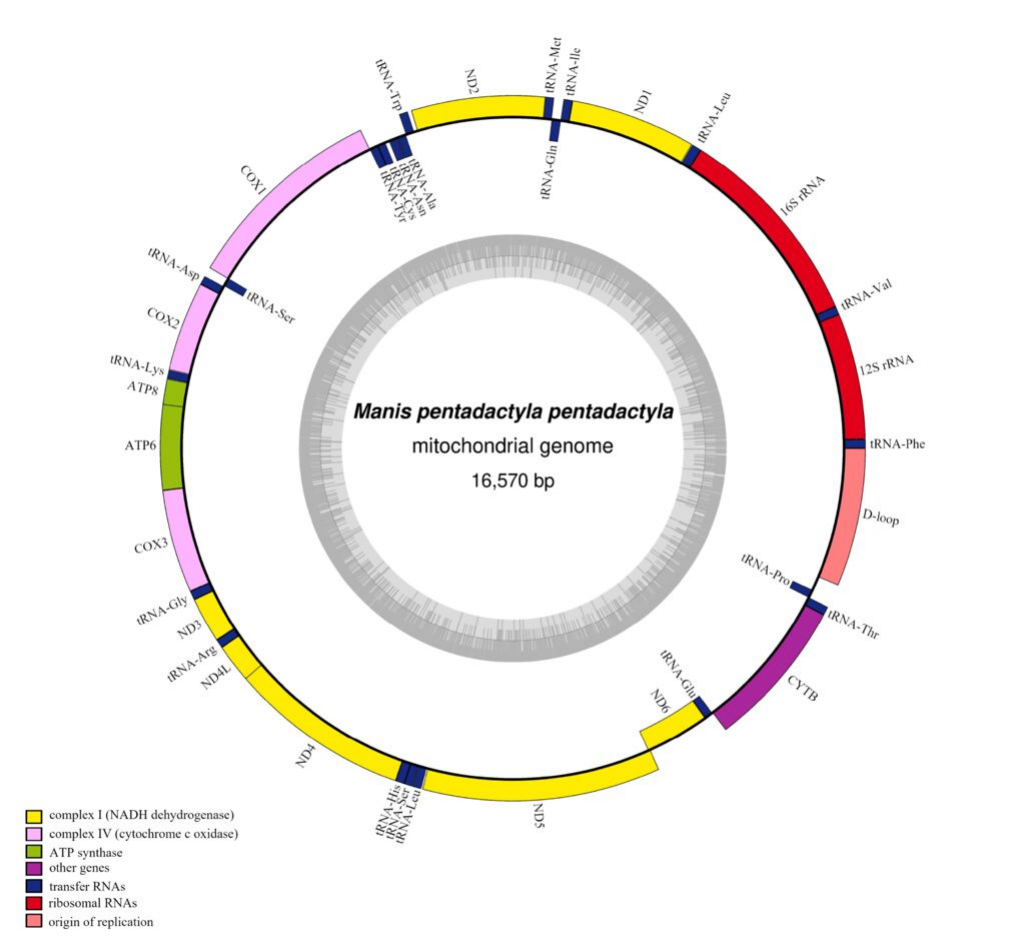
Complete mitochondrial genome map of *Manispentadactylapentadactyla*. The grey small circle represents GC content graph.

The mitogenome sequence of *M.p.pentadactyla* was 16,570 bp in length and contained 22 tRNAs, two rRNAs and 13 PCGs (Fig. 1, Table 1). The mitogenome size was well within the range found in Manidae, from 16540 (*Smutsiagigantean*, GenBank No. MF536684) to 16,577 bp (*Manispentadactyla*, KT445978) (Suppl. material 1). The gene order, gene identity and gene number were consistent with those of pangolins mitogenomes (Du Toit et al. 2017). The light and heavy strands each contain their own arrangement of genes, proteins or loci. The 14 tRNA, two rRNA and 12 PCGs were located in the heavy strand and other genes in the light strand (Fig. 1, Table 2). The frequencies of adenine (A), cytosine (C), guanine (G) and thymine (T) were 34.5%, 26.9%, 13.2% and 25.4%, respectively. In addition, the nucleotide composition of the mitogenome was slightly A + T biased, which is similar to that of the other pangolins (Suppl. material 1) (Du Toit et al. 2017, Hua et al. 2020). These results also confirmed the A + T bias, which has been reported in several other mammalian species (Dou et al. 2016). The skew statistics of the whole genome showed that the whole mitochondrial genome of *M.p.pentadactyla* is AT skewed (0.1515) and GC skewed (-0.3406). Positive AT skew and negative GC skew were also found in the other pangolins (Suppl. material 1).

### Protein-coding genes and codon usages

The sequence length of 13 PCGs was 11,396 bp with base compositions of 31.84%, 29.35%, 13.28% and 25.53% for A, C, G and T, respectively (Table 2). The shortest gene was ATP8 (201 bp) and the longest gene was ND5 (1,812 bp). The A + T content of 13 PCGs ranged from 52.68% (COX3) to 60.06% (ND2), with an average of 57.37%, showing a slight A + T bias. According to the AT skew and GC skew analyses, all PCGs exhibited a stronger nucleotide asymmetry with a positive AT skew, but negative GC skew, except for ND6 (Table 2). Analysis of codon usage of most PCGs provided evidence of bias in terms of the use of codons, with A and C occurring most frequently. The asymmetrical base composition observed in the PCGs of the *M.p.pentadactyla* mitogenomes was probably due to the process of mutation and/or adaptive selection (Mooers and Holmes 2000, Castellana et al. 2011).

All PCGs started with a typical ATN codon: four (ND1, ND2, COX1, COX2, ATP8, ATP6, COX3, ND4L, ND4, ND6 and CYTB) with ATG and two (ND3 and ND5) with ATA. The complete stop codons, TAA and AGA, were found in six genes (ND1, COX2, ATP8, ATP6, ND4L and ND5) and three genes (COX1, ND6 and CYTB), while the remaining five genes terminated with a single base T (ND2, COX3, ND4) or one base TA (ND3) (Table 1).

### Transfer and ribosomal RNA

**Figure 2.**
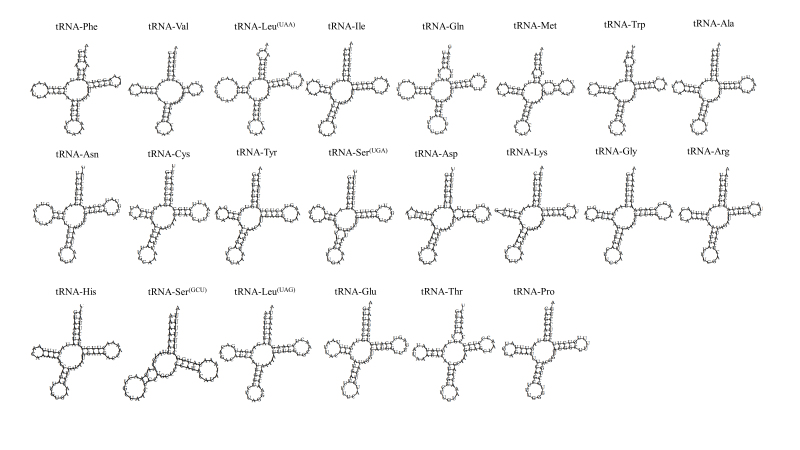
Putative secondary structure of the 22 tRNAs of *Manispentadactylapentadactyla*. The tRNAs are labelled with the abbreviations of their corresponding amino acids.

The sequence lengths of 12S and 16S rRNA were 960 bp and 1,566 bp, respectively. The base composition of both rRNAs was 37.92% A, 21.54% C, 17.74% G and 22.80% T and the A + T content was 60.72%. The 22 tRNAs were interspersed in the whole mitochondrial genome, varying from 59 (tRNA-Ser^(GCU)^) to 73 nucleotides (tRNA-Gln and tRNA-Asn). The A + T content of these tRNAs was 63.68% and positive AT skew and negative GC skew were found (Table 2). The predicted secondary structures of the 22 tRNAs are shown in Fig. 2. All tRNAs were folded into typical cloverleaf-shaped secondary structures which included an amino acid accepting arm, DHU loop, anticodon loop and TψC loop, except for tRNA-Ser^(GCU)^. The tRNA-Ser^(GCU)^ lacks the DHU loop arm stem and DHU loop. Missing the DHU loop arm stem and DHU loop for tRNA-Ser^(GCU)^ is common in metazoan mitochondria (Jühling et al. 2009, Watanabe et al. 2014).

### Phylogenetic analysis

**Figure 3.**
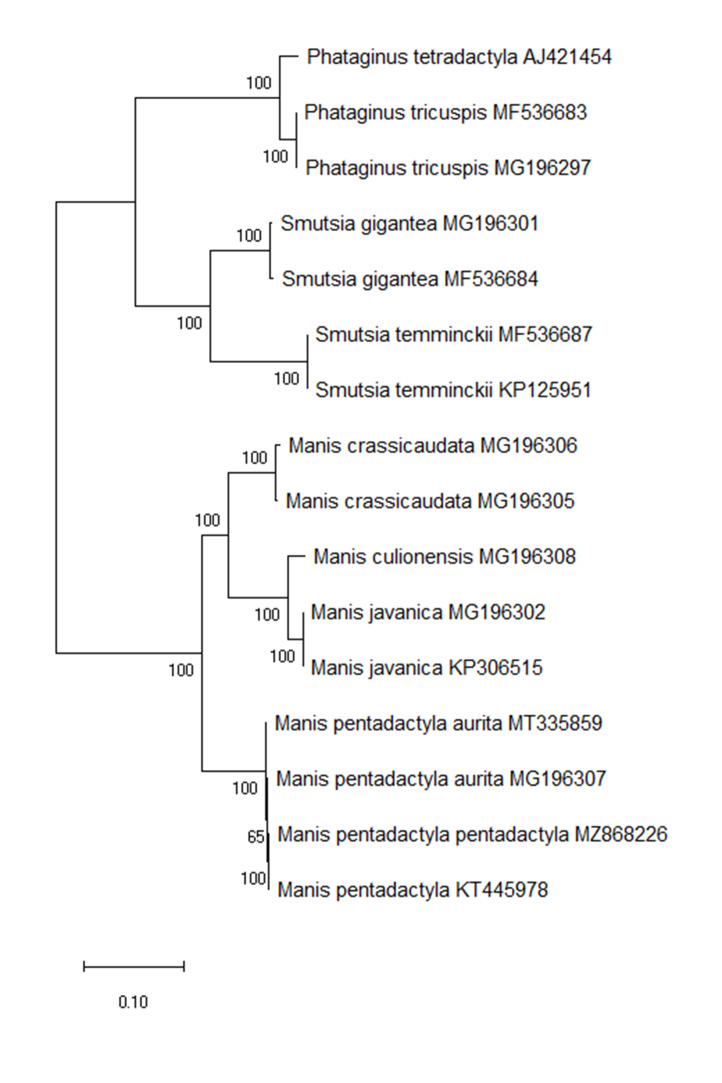
Maximum Likelihood (ML) phylogenetic tree of *Manispentadactylapentadactyla* and other pangolins. The numbers on branch lengths are bootstrap values.

The topology of phylogenetic trees, based on full mitogenome sequences, illustrated that Asian and African pangolin species were separated into two distinct monophyletic clades, consistent with previous studies (Gaubert et al. 2018). *Manisp.pentadactyla* and all *M.p.aurita* were clustered in the same clade, but *M.p.aurita* was a paraphyletic group (Fig. 3). We found that *M.p.pentadactyla* was closely related to *M.pentadactyla* KT445978, with 98.92% nucleotide similarity within the same clade. The sequence (KT445978) was only labelled as *M.pentadactyla* mtDNA sequence from NCBI and geographic location of this sample was Taiwan. The results of phylogenetic analysis indicated the individual (KT445978) was identified as *M.p.pentadactyla* and *M.p.pentadactyla* was a monophyletic group (Fig. 3). For the combined 13 PCG sequences, the phylogeny was also consistent with full mitogenome sequences. Funk and Omland (2003) reviewed the phylogenetic studies of mitochondrial DNA in animals, indicating that 23% of species were reconstructed as paraphyletic or polyphyletic relationships. Paraphyletic or polyphyletic groups are common in nature, even seen in plants (Hörandl 2006, Hörandl and Stuessy 2010). The mitogenome phylogeny of Eurasian lynx (*Lynxlynx*) indicated that three subspecies (*L.l.lynx*, *L.l.dinniki* and *L.l.wrangeli*) are paraphyletic groups (Mengüllüoğlu et al. 2021). The phylogeny of the paraphyletic group resulted from the stochasticity of the coalescent process, which is more likely to result in larger population sizes or shorter divergence times, leading to incomplete lineage sorting (Pamilo and Nei 1988, Díaz et al. 2018). However, introgression or hybridisation is another biological factor that causes this phenomenon (Harrison and Larson 2014). The current hybridisation between both subspecies is less possible due to the geographical barrier (Taiwan Strait) and the paraphyly of *M.p.aurita* is likely a result of incomplete lineage sorting. Geographic isolation is known to contribute to divergent evolution, resulting in the monophyly of *M.p.pentadactyla* restricted to Taiwan. The results provide important clues for understanding the phylogeny of the three subspecies of *M.pentadactyla*. Furthermore, additional sampling of *M.p.pentadactyla* and *M.p.pusilla* will help us to clarify the phylogeny.

## Conclusions

The complete mitochondrial genome of *M.p.pentadactyla* was sequenced and the mitogenome sequence was 16,570 bp in size. The AT content was higher than the GC content, which is consistent with the findings of other pangolins. Maximum Likelihood phylogenetic analysis of the complete mitogenome and 13 PCGs showed that *M.p.pentadactyla* and all *M.p.aurita* were clustered in the same clade and *M.p.pentadactyla* was a monophyletic group, but *M.p.aurita* was a paraphyletic group. The complete mitogenome of *M.p.pentadactyla*, reported in this study, enriches the mitogenome database of the Chinese pangolin and provides useful information for the phylogeny and taxonomy of *M.pentadactyla*. Based on our study, to identify subspecies of the Chinese pangolins using mtDNA markers in the wildlife trade of the Chinese pangolin can still be difficult. The Chinese pangolins have a wide range of distribution, within which intraspecific divergences may occur in some regions, especially in west of Myanmar, north and northeast India, Bangladesh, Bhutan and Nepal. To clearly confirm the status of the subspecies of the Chinese pangolin and address the conservation efforts of wildlife trade, further studies are needed, especially for the subspecies (*M.p.pusilla*) on Hainan Island, along with greater geographic sampling of *M.p.aurita* and *M.p.pendactyla*.

## Data resources

The genome sequence data are available in GenBank (https://www.ncbi.nlm.nih.gov/) under accession no. MZ868226.

## Supplementary Material

AAE8DDB0-6C30-5BB7-80B3-13763AB39F14 10.3897/BDJ.9.e77961.suppl1 Supplementary material 1 Mitochondrial genome characteristics Data type Table Brief description Characteristics of the mitochondrial genome of pangolins used in this study. File: oo_625289.pdf https://binary.pensoft.net/file/625289 Kuo-Hsiang Hung

## Figures and Tables

**Table 1.** Characteristics of the mitochondrial genome of *Manispentadactylapentadactyla*.

Gene	Position		Length (bp)	Anticodon	Codon		Intergenic nucleotides*	Strand
	From	To			Start	Stop		
tRNA-Phe	1	68	68	GAA			0	H
12S ribosomal RNA	69	1028	960				0	H
tRNA-Val	1028	1093	66	UAC			-1	H
16S ribosomal RNA	1094	2659	1566				0	H
tRNA-Leu ^(UAA)^	2660	2733	74	UAA			0	H
ND1	2737	3693	957		ATG	TAA	3	H
tRNA-Ile	3693	3761	69	GAU			-1	H
tRNA-Gln	3759	3831	73	UUG			-3	L
tRNA-Met	3833	3901	69	CAU			1	H
ND2	3875	4913	1039		ATG	T--	-27	H
tRNA-Trp	4941	5007	67	UCA			27	H
tRNA-Ala	5011	5079	69	UGC			3	L
tRNA-Asn	5081	5153	73	GUU			1	L
tRNA-Cys	5186	5250	65	GCA			32	L
tRNA-Tyr	5251	5317	67	GUA			0	L
COX1	5319	6869	1551		ATG	AGA	1	H
tRNA-Ser^(UGA)^	6865	6933	69	UGA			-5	L
tRNA-Asp	6941	7007	67	GUC			7	H
COX2	7008	7691	684		ATG	TAA	0	H
tRNA-Lys	7694	7757	64	UUU			2	H
ATP8	7759	7959	201		ATG	TAA	1	H
ATP6	7920	8600	681		ATG	TAA	-40	H
COX3	8600	9383	784		ATG	T--	-1	H
tRNA-Gly	9384	9452	69	UCC			0	H
ND3	9453	9799	347		ATA	TA-	0	H
tRNA-Arg	9800	9866	67	UCG			0	H
ND4L	9867	10163	297		ATG	TAA	0	H
ND4	10157	11534	1378		ATG	T--	-7	H
tRNA-His	11535	11602	68	GUG			0	H
tRNA-Ser ^(GCU)^	11603	11661	59	GCU			0	H
tRNA-Leu ^(UAG)^	11663	11733	71	UAG			1	H
ND5	11743	13554	1812		ATA	TAA	9	H
ND6	13538	14062	525		ATG	AGA	-17	L
tRNA-Glu	14063	14131	69	UUC			0	L
CYTB	14135	15274	1140		ATG	AGA	3	H
tRNA-Thr	15275	15341	67	UGU			0	H
tRNA-Pro	15341	15407	67	UGG			-1	L
D_loop	15521	16570	1049				113	H

Notes: * The numbers of nucleotides between the given and its previous gene, negative values indicate an overlap; H indicated that the genes are transcribed on the heavy strand.

**Table 2.** Nucleotide composition in two rRNA, 13 protein-coding genes and 22 tRNA of mitochondrial genome of *Manispentadactylapentadactyla*.

	Adenine (%)	Cytosine (%)	Guanine (%)	Thymine (%)	A+T (%)	AT skew	GC skew	Length (bp)
**ribosomal RNA**	37.92	21.54	17.74	22.80	60.72	0.2490	-0.0967	2,526
12S ribosomal RNA	36.87	22.92	17.92	22.29	59.16	0.2465	-0.1224	960
16S ribosomal RNA	38.57	20.69	17.62	23.12	61.69	0.2504	-0.0801	1,566
**protein-coding genes**	31.84	29.35	13.28	25.53	57.37	0.1100	-0.3770	11,396
ND1	33.02	29.99	12.23	24.76	57.78	0.1430	-0.4207	957
ND2	37.63	30.80	9.14	22.43	60.06	0.2531	-0.5423	1,039
COX1	28.43	27.21	15.51	27.85	56.28	0.0103	-0.2739	1,551
COX2	32.16	28.95	13.74	25.15	57.31	0.1223	-0.3563	684
ATP8	38.81	29.85	7.46	23.88	62.69	0.2382	-0.6001	201
ATP6	33.33	32.89	10.72	23.06	56.39	0.1821	-0.5084	681
COX3	26.79	30.99	16.33	25.89	52.68	0.0171	-0.3098	784
ND3	34.29	31.70	10.95	23.06	57.35	0.1958	-0.4865	347
ND4L	31.98	29.97	11.45	26.60	58.58	0.0918	-0.4471	297
ND4	33.67	31.57	11.25	23.51	57.18	0.1777	-0.4745	1,378
ND5	34.93	30.41	9.88	24.78	59.71	0.1700	-0.5096	1,812
ND6	16.38	8.57	32.57	42.48	58.86	-0.4434	0.5834	525
CYTB	30.53	31.67	13.86	23.94	54.47	0.1210	-0.3912	1,140
**transfer DNA**	36.46	20.94	15.38	27.22	63.68	0.1451	-0.1531	1495

## References

[B7530163] Allen G. M. (1938). The mammals of China and Mongolia. Natural History of Central Asia.

[B7530145] Allen J. A. (1906). Mammals from the Island of Hainan, China. Bulletin of the American Museum of Natural History.

[B7530171] Allio R., Schomaker-Bastos A., Romiguier J., Prosdocimi F., Nabholz B., Delsuc F. (2020). MitoFinder: Efficient automated large-scale extraction of mitogenomic data in target enrichment phylogenomics. Molecular Ecology Resources.

[B7531155] Castellana S., Vicario S., Saccone C. (2011). Evolutionary patterns of the mitochondrial genome in metazoa: exploring the role of mutation and selection in mitochondrial protein-coding genes. Genome Biology and Evolution.

[B7531181] Challender D., Wu S. B., Kaspal P., Khatiwada A., Ghose A., Sun N. C.M., Mohapatra R. K., Suwal T. (2019). *Manispentadactyla* (errata version published in 2020). The IUCN Red List of Threatened Species 2019: e.T12764A168392151..

[B7531164] Challender D. W.S., Waterman C., Baillie J. E.M. (2014). Scaling up pangolin conservation. IUCN SSC Pangolin Specialist Group Conservation Action Plan.

[B7531272] Chao J. T., H.F. Li., Lin C. C., Challender D. W.S., Nash H. C., Waterman C. (2020). Pangolins: Science, Society and Conservation.

[B7533911] Chen S., Zhou Y., Chen Y., Gu J. (2018). fastp: an ultra-fast all-in-one FASTQ preprocessor. Bioinformatics.

[B7531285] CITES (2021). Appendixes I, II and III. https://cites.org/eng/app/appendices.php.

[B7531293] Del Toro I., Ribbons R. R., Pelini S. L. (2012). The little things that run the world: a review of ant mediated ecosystem services and disservices (Hymenoptera: Formicidae). Myrmecological News.

[B7532827] Díaz F., Lima A. L.A., Nakamura A. M., Fernandes F., Sobrinho I. Jr., Brito R. A. (2018). Evidence for introgression among three species of the *Anastrephafraterculus* group, a radiating species complex of fruit flies. Frontiers in Genetics.

[B7532850] Donath A., Jühling F., Al-Arab M., Bernhart S. H., Reinhardt F., Stadler P. F., Middendorf M., Bernt M. (2019). Improved annotation of protein-coding genes boundaries in metazoan mitochondrial genomes. Nucleic Acids Research.

[B7532868] Dou H., Zhang Y., Feng L. (2016). Complete mitochondrial genome of the Himalayan serow (*Capricornisthar*) and its phylogenetic status within the genus *Capricornis*. Biochemical Systematics and Ecology.

[B7533929] Du Toit Z., Du Plessis M., Dalton D. L., Jansen R., Grobler P. J., Kotzé A. (2017). Mitochondrial genomes of African pangolins and insights into evolutionary patterns and phylogeny of the family Manidae. BMC Genomics.

[B7532928] Funk D. J., Omland K. E. (2003). Species level paraphyly and polyphyly: Frequency, causes, and consequences, with insights from animal mitochondrial DNA. Annual Review of Ecology, Evolution, and Systematics.

[B7619195] Gaubert P., Antunes A., Meng H., Miao L., Peign&eacute S., Justy F., Njiokou F., Dufour S., Danquah E., Alahakoon J., Verheyen E., Stanley W. T., O'Brien S. J., Johnson W. E., Luo S. J. (2018). The complete phylogeny of pangolins: Scaling up resources for the molecular tracing of the most trafficked mammals on earth. Journal of Heredity.

[B7532937] Harrison J. L. (1961). The natural food of some Malayan mammals. Bulletin of the Singapore National Museum.

[B7532947] Harrison R. G., Larson E. L. (2014). Hybridization, introgression, and the nature of species boundaries. Journal of Heredity.

[B7533940] Hörandl E. (2006). Paraphyletic versus monophyletic taxa - evolutionary versus cladistic classifications. Taxon.

[B7533949] Hörandl E., Stuessy T (2010). Paraphyletic groups as natural units of biological classification. Taxon.

[B7532979] Hua Y., Wang J., An F., Xu J., Zhang H., Gu H. (2020). Phylogenetic relationship of Chinese pangolin (*Manispentadactylaaurita*) revealed by complete mitochondrial genome. Mitochondrial DNA Part B.

[B7533000] Jühling F., Mörl M., Hartmann R. K., Sprinzl M., Stadler P. F., Pütz J. (2009). tRNAdb 2009: compilation of tRNA sequences and tRNA genes. Nucleic Acids Research.

[B7533051] Katoh K., Rozewicki J., Yamada K. D. (2019). MAFFT online service: multiple sequence alignment, interactive sequence choice and visualization. Briefings in Bioinformatics.

[B7533040] Kumar S., Stecher G., Li M., Knyaz C., Tamura K. (2018). MEGA X: molecular evolutionary genetics analysis across computing platforms. Molecular Biology and Evolution.

[B7533282] Lee R. H., Cheung K., Fellowes J. R., Guénard B. (2017). Insights into the Chinese pangolin’s (*Manispentadactyla*) diet in a peri-urban habitat: A case study from Hong Kong. Tropical Conservation Science.

[B7533313] Lohse M., Drechsel O., Bock R. (2007). OrganellarGenomeDRAW (OGDRAW): a tool for the easy generation of high-quality custom graphical maps of plastid and mitochondrial genomes. Current Genetics.

[B7533920] Magoč T., Salzberg S. (2011). FLASH: fast length adjustment of short reads to improve genome assemblies. Bioinformatics.

[B7533330] Mengüllüoğlu D., Ambarlı H., Barlow A., Paijmans J. L.A., Sayar A. O., Emir H., Kandemir İ., Hofer H., Fickel J., Förster D. W. (2021). Mitogenome phylogeny including data from additional subspecies provides new insights into the historical biogeography of the Eurasian lynx
*Lynxlynx*. Genes.

[B7619980] Minh B. Q., Nguyen M. A.T., Haeseler A. (2013). Ultrafast approximation for phylogenetic bootstrap. Molecular Biology and Evolution.

[B7533441] Mooers A. Ø., Holmes E. C. (2000). The evolution of base composition and phylogenetic inference. Trends in Ecology & Evolution.

[B7619971] Nguyen L. T., Schmidt H., Von Haeseler A., Minh B. (2015). IQ-TREE: a fast and effective stochastic algorithm for estimating maximum-likelihood phylogenies. Molecular Biology and Evolution.

[B7533477] Pamilo P., Nei M. (1988). Relationships between gene trees and species trees. Molecular Biology and Evolution.

[B7533486] Perna N., Koeher T. (1995). Patterns of nucleotide composition at fourfold degenerate sites of animal mitochondrial genomes. Journal of Molecular Evolution.

[B7533504] Pietersen D. W., Symes C. T., Woodborne S., McKechnie A. E., Jansen R. (2016). Diet and prey selectivity of the specialist myrmecophage, Temminck’s ground pangolin. Journal of Zoology.

[B7533532] Pietersen D. W., Challender D. W.S., Challender D. W.S., Nash H. C., Waterman C. (2020). Pangolins: Science, society and conservation.

[B7533546] Sambrook J., Russell D. W. (2001). Molecular cloning, a laboratory manual.

[B7533558] Sun N. C.M., Arora B., Lin J. S., Lin W. C., Chi M. J., Chen C. C., Pei K. J.C. (2019). Mortality and morbidity in wild Taiwanese pangolin (*Manispentadactylapentadactyla*). PLOS One.

[B7533570] Sun N. C.M., Chang S. P., Lin J. S., Tseng Y. W., Pei K. J.C., Hung K. H. (2020). The genetic structure and mating system of a recovered Chinese pangolin (*Manispentadactyla* Linnaeus, 1758) population as inferred by microsatellite markers. Global Ecology and Conservation.

[B7533598] Watanabe Y., Suematsu T., Ohtsuki T. (2014). Losing the stem-loop structure from metazoan mitochondrial tRNAs and co-evolution of interacting factors. Frontiers in Genetics.

[B7533611] Wu S. B., Sun N. C.M., Zhang F. H., Yu Y. S., Ades G., Suwal T. L., Jiang Z., Challender D. W.S, Nash H. C., Waterman C. (2020). Pangolins: Science, society and conservation.

[B7533585] Wu S. H., Chen M., Chin S. C., Lee D. J., Wen P. Y., Chen L. W., Wang B. T., Yu H. T. (2007). Cytogenetic analysis of the Formosan pangolin, *Manispentadactylapentadactyla* (Mammalia: Pholidota). Zoological Studies.

