# Supplementary material for: Complete mitochondrial genome of Manispentadactylapentadactyla (Mammalia: Pholidota), an endemic subspecies of Chinese pangolin: mitogenome characterisation and phylogenetic implications
Source: Biodivers Data J. 2021 Dec 29;9:e77961. doi: 10.3897/BDJ.9.e77961 (PMC8732882; doi:10.3897/BDJ.9.e77961)
Supplement: Supplementary material 1 — Mitochondrial genome characteristics [file bdj-09-e77961-s001.pdf]

Supplementary material 1. Characteristics of the mitochondrial genome of pangolins used in this study.

species	Accession Number	Length (bp)	A+T (%)	G+C (%)	AT–skew	GC–skew	reference
Manis pentadactyla							
	KT445978	16,577	59.89	40.11	0.1513	-0.3410	Hari et al. (2016)
Manis pentadactyla aurita							
	MG196307	16,573	59.86	40.14	0.1510	-0.3392	Gaubert et al. (2017)
	MT335859	16,573	59.93	40.07	0.1512	-0.3402	Hua et al. (2020)
Manis crassicaudata							
	MG196305	16,575	57.64	42.36	0.1668	-0.3377	Gaubert et al. (2017)
	MG196306	16,576	57.70	42.30	0.1657	-0.3362	Gaubert et al. (2017)
Manis javanica							
	MG196302	16,578	56.15	43.85	0.1786	-0.3298	Gaubert et al. (2017)
	KP306515	16,574	56.16	43.84	0.1786	-0.3295	Hassanin et al. (2015)
Manis culionensis							
	MG196308	16,577	56.39	43.61	0.1781	-0.3320	Gaubert et al. (2017)
Smutsia gigantea							
	MG196301	16,548	63.11	36.89	0.1028	-0.3172	Gaubert et al. (2017)
	MF536684	16,540	63.05	36.95	0.1029	-0.3163	Du Toit et al. (2017)
Smutsia temminckii							
	MF536687	16,558	62.72	37.28	0.0844	-0.2872	Du Toit et al. (2017)
	KP125951	16,559	62.73	37.27	0.0844	-0.2874	Du Toit et al. (2014)
Phataginus tetradactyla							
	AJ421454	16,571	63.06	36.94	0.0564	-0.2565	Arnason et al. (2002)
Phataginus tricuspis							
	MF536683	16,565	63,26	36,74	0.0532	-0.2521	Du Toit et al. (2017)
	MG196297	16,564	63.29	36.71	0.0534	-0.2520	Gaubert et al. (2017)

Reference:

Arnason U, Adegoke JA, Bodin K, Born EW, Esa YB, Gullberg A, Nilsson M, Short RV, Xu X, Janke A (2002) Mammalian mitogenomic relationships and the root of the eutherian tree. Proceedings of the National Academy of Sciences of the United States of America 99: 8151–8156. https://doi.org/10.1073/pnas.102164299

Du Toit Z, Du Plessis M, Dalton DL, Jansen R, Grobler PJ, Kotzé A (2017) Mitochondrial genomes of African pangolins and insights into evolutionary patterns and phylogeny of the family Manidae. BMC Genomics 18: 746-758. https://doi.org/10.1186/s12864-017-4140-5

Gaubert P, Antunes A, Meng H, Miao L, Peign&eacute S, Justy F, Njiokou F, Dufour S, Danquah E, Alahakoon J, Verheyen E, Stanley WT, O'Brien SJ, Johnson WE, Luo SJ (2018) The complete phylogeny of pangolins: Scaling up resources for the molecular tracing of the most trafficked mammals on earth. Journal of Heredity 109(4):347-359 https://doi.org/10.1093/jhered/esx097

Hari R, Paterson IC, Choo SW (2016) A new complete mitogenome of the critically endangered Chinese pangolin *Manis pentadactyla*. Conservation Genetics Resources 8: 423–426. https://doi.org/10.1007/s12686-016-0586-z

Hassanin A, Hugot JP, van Vuuren BJ (2015) Comparison of mitochondrial genome sequences of pangolins (Mammalia, Pholidota). Comptes Rendus Biologies 338(4):260-265. https://doi.org/10.1016/j.crvi.2015.02.003

Hua Y, Wang J, An F, Xu J, Zhang H, Gu H (2020) Phylogenetic relationship of Chinese pangolin (*Manis pentadactyla aurita*) revealed by complete mitochondrial genome. Mitochondrial DNA Part B 5 (3): 2523-2524. https://doi.org/10.1080/23802359.2020.1772693
